# Association Between Catenin Beta-1 (CTNNB1) Gene Polymorphisms and Non-Traumatic Osteonecrosis of the Femoral Head (ONFH)

**DOI:** 10.3390/cimb48020164

**Published:** 2026-02-01

**Authors:** I-Chang Lai, De-Yi Liu, Shih-Chan Hsu, Shu-Jui Kuo

**Affiliations:** 1 Department of Education, China Medical University Hospital, Taichung 404327, Taiwan; lai.i.chang.58@gmail.com (I.-C.L.); deyil845@gmail.com (D.-Y.L.); 2 School of Medicine, China Medical University, Taichung 404328, Taiwan; jamieee0424@gmail.com; 3 Department of Orthopedic Surgery, China Medical University Hospital, Taichung 404327, Taiwan

**Keywords:** CTNNB1 (catenin beta-1) gene, genetic polymorphisms, osteonecrosis of the femoral head (ONFH), single-nucleotide polymorphisms (SNPs)

## Abstract

Non-traumatic osteonecrosis of the femoral head (ONFH) is a multifactorial disorder in which genetic susceptibility is thought to play an important role, yet the contribution of many candidate genes remains unclear. The catenin beta-1 (CTNNB1) gene encodes β-catenin, a key regulator of the Wnt/β-catenin signaling pathway involved in bone homeostasis and vascular regulation, and may therefore influence susceptibility to non-traumatic ONFH. In this case–control study, genotype data from China Medical University Hospital were analyzed to evaluate the association between CTNNB1 polymorphisms and the risk of ONFH. A total of 609 patients with ONFH and 2436 age- and sex-matched controls were included. Fourteen CTNNB1 single-nucleotide polymorphisms (SNPs) with a minor allele frequency greater than 5% were selected and analyzed using logistic regression under multiple genetic models, with Hardy–Weinberg equilibrium assessed in controls. Two SNPs, rs3774370 and rs11564478, showed significant differences in allele frequencies between cases and controls, with lower minor allele frequencies observed in the ONFH group. Both variants were associated with a reduced risk of ONFH, and these associations remained significant under dominant genetic models. These findings suggest that specific CTNNB1 polymorphisms may confer a protective effect against non-traumatic ONFH and provide further insight into the genetic architecture of this disease.

## 1. Introduction

Non-traumatic osteonecrosis of the femoral head (ONFH) is a progressive and debilitating orthopedic disorder characterized by bone tissue necrosis secondary to compromised blood supply, occurring in the absence of direct mechanical injury. It represents a complex disease entity encompassing distinct clinical stages, heterogeneous pathogenic mechanisms, and evolving diagnostic and therapeutic strategies, and remains a major cause of hip joint destruction and subsequent arthroplasty worldwide [1,2,3,4,5,6,7,8,9]. The disease predominantly affects young to middle-aged adults and accounts for approximately 10% of total hip arthroplasties performed annually in the United States, resulting in substantial functional impairment and socioeconomic burden [9]. Unlike traumatic ONFH, which arises from acute disruption of femoral head vascularity following fracture or dislocation, non-traumatic ONFH is closely associated with systemic and metabolic factors, most notably corticosteroid exposure and excessive alcohol consumption, which together account for more than 80% of cases [9]. Nevertheless, only a subset of exposed individuals develops non-traumatic ONFH, and some patients present without identifiable risk factors, strongly implicating genetic susceptibility in disease pathogenesis [10].

Single-nucleotide polymorphisms (SNPs) are the most common form of genetic variation and can influence gene expression, protein function, and downstream biological pathways, thereby modulating individual susceptibility to complex diseases [11,12,13,14,15,16,17,18,19,20,21,22,23,24,25,26,27,28,29,30,31,32,33,34,35,36,37,38,39,40,41,42,43,44,45]. Increasing evidence suggests that genetic factors play a critical role in non-traumatic ONFH, particularly through pathways involved in lipid metabolism, vascular homeostasis, inflammation, and bone remodeling [10]. For example, polymorphisms in the RAB40C gene have been associated with alcohol-induced non-traumatic ONFH, potentially through dysregulated lipid metabolism and impaired bone homeostasis [46]. Variants in the nitric oxide synthase 3 (NOS3) gene, which encodes endothelial nitric oxide synthase, have been linked to altered vascular function and reduced susceptibility to non-traumatic ONFH [47]. In addition, SNPs in genes such as phosphofructokinase, platelet (PFKP) and glypican 6 (GPC6) have been correlated with alcohol-induced ONFH, while polymorphisms in inflammatory mediators including TNFRSF11A, IL-6, and TNF have also been implicated in disease susceptibility [48,49,50]. Despite these advances, no single genetic variant has demonstrated robust predictive power, underscoring the polygenic and multifactorial nature of non-traumatic ONFH.

In the current era of genome-wide association studies (GWAS), hypothesis-free approaches have substantially expanded the identification of genetic loci associated with complex diseases [45,51,52,53,54,55]. However, GWAS findings often explain only a limited proportion of disease heritability and may highlight loci with uncertain biological relevance [52,56]. Consequently, candidate gene studies guided by established disease pathogenesis remain of considerable value, particularly for conditions such as non-traumatic ONFH, in which specific biological mechanisms—namely vascular compromise, impaired osteogenesis, and aberrant mesenchymal stem cell differentiation—are well recognized. Pathogenesis-driven analyses can complement GWAS by prioritizing biologically plausible genes, facilitating mechanistic interpretation, and enhancing translational relevance [57,58,59].

The catenin beta-1 (CTNNB1) gene encodes β-catenin, a central component of the canonical Wnt/β-catenin signaling pathway, which regulates cellular proliferation, differentiation, and tissue homeostasis. Activation of this pathway involves binding of Wnt ligands to Frizzled receptors and low-density lipoprotein-related receptors 5 and 6 (LRP5/6), resulting in stabilization and nuclear translocation of β-catenin. In the nucleus, β-catenin interacts with T-cell factor/lymphoid enhancer factor (TCF/LEF) transcription factors to regulate the expression of osteogenic genes such as Runx2 and osteocalcin, both of which are essential for skeletal development and bone remodeling [60]. Dysregulation of Wnt/β-catenin signaling has been implicated in impaired angiogenesis, reduced osteogenic differentiation, and enhanced adipogenesis of mesenchymal stem cells—hallmark features of non-traumatic ONFH pathophysiology [60,61,62].

Genetic polymorphisms in CTNNB1 have been associated with susceptibility to multiple human diseases, including prostate, gastric, breast, ovarian, colorectal, and hepatocellular cancers [63], highlighting the biological importance of subtle variations in β-catenin signaling. Despite the well-established role of CTNNB1 in bone metabolism and vascular regulation, and its relevance to key mechanisms underlying non-traumatic ONFH, the association between CTNNB1 genetic polymorphisms and susceptibility to non-traumatic ONFH has not yet been explored.

Accordingly, the present study aimed to investigate the association between CTNNB1 gene polymorphisms and the risk of non-traumatic osteonecrosis of the femoral head using a hospital-based case–control design. By focusing on a biologically relevant candidate gene within a core pathogenic pathway, this study seeks to clarify the contribution of CTNNB1 to non-traumatic ONFH susceptibility and to further elucidate the genetic architecture of this complex disease.

## 2. Materials and Methods

Genotype and clinical data were obtained from China Medical University Hospital (CMUH), Taiwan. All individuals aged 20 years or older who had visited CMUH since January 2003 and had provided written informed consent for genetic research were eligible for inclusion. In addition to routine clinical blood sampling, up to 10 mL of peripheral venous blood was collected from each participant for research purposes. Clinical diagnoses were coded using the International Classification of Diseases, Ninth Revision (ICD-9) and Tenth Revision (ICD-10).

Patients with ONFH were identified based on ICD-9 code 733.42 and/or ICD-10 code M87.05 recorded between January 2003 and January 2023. These diagnostic codes were assigned by attending physicians in outpatient clinics, emergency departments, or inpatient wards and subsequently verified during submission to Taiwan’s National Health Insurance system. To focus on non-traumatic ONFH, records associated with acute trauma-related diagnoses, including hip fracture (ICD-9 820.X, ICD-10 S72.0, S72.1) or hip dislocation (ICD-9 835.X, ICD-10 S73.0), were excluded. Individuals without any diagnostic coding indicative of ONFH were defined as control subjects. Based on this case ascertainment strategy, the study cohort predominantly represents non-traumatic ONFH.

Each ONFH case was matched with four control subjects according to sex (male or female) and age group (≤40 years or >40 years), yielding a 1:4 case–control ratio. The study protocol was approved by the Institutional Review Board of CMUH (approval number: CMUH112-REC3-014).

Single-nucleotide polymorphism (SNP) genotyping was performed using the Taiwan Biobank version 2 (TWBv2) custom array, developed by Thermo Fisher Scientific (Santa Clara, CA, USA). The TWBv2 array was designed to capture both genome-wide association study (GWAS) markers and functional genetic variants, providing comprehensive coverage of common and rare variants relevant to disease susceptibility. The array contains approximately 690,000 markers aligned to the GRCh38 reference genome, including more than 100,000 coding variants and nearly 93,000 protein-altering variants, enabling high-resolution genetic analysis [64,65].

Fourteen SNPs located within or in close proximity to the CTNNB1 gene were selected for analysis based on their availability on the Taiwan Biobank version 2 (TWBv2) array and genomic coverage of the CTNNB1 locus. Minor allele frequencies (MAFs) were subsequently calculated in the study population and are reported accordingly. Both common and low-frequency variants were included to comprehensively assess genetic variation within the CTNNB1 gene region. Genomic locations and variant annotations were referenced from the National Center for Biotechnology Information (NCBI) database.

Statistical analyses were conducted using PLINK software (version 1.; Purcell Lab, Boston, MA, USA 9) and R statistical software (version 4.4.2; R Foundation for Statistical Computing, Vienna, Austria). Continuous variables were compared using Student’s *t*-test or analysis of variance (ANOVA), as appropriate. The association between CTNNB1 SNPs and susceptibility to ONFH was evaluated using logistic regression analysis, with odds ratios (ORs) and 95% confidence intervals (CIs) calculated to estimate effect size. Statistical significance was defined as a two-sided *p* value < 0.05. Adjusted logistic regression analyses included age and sex as covariates, consistent with the matching factors used in the case–control study design.

Hardy–Weinberg equilibrium (HWE) was assessed in the control group using the chi-square test as a genotyping quality control measure to evaluate potential deviations from expected genotype frequencies. HWE testing was applied to detect possible genotyping errors and does not replace formal assessment of population stratification. SNPs showing extreme deviation from HWE in controls (*p* < 1 × 10^−6^) were excluded. In the present study, no SNP met this exclusion criterion. rs3774371 showed a borderline deviation from HWE (*p* = 0.029); however, it was retained because it was not associated with ONFH risk and did not affect the main findings.

Multiple genetic inheritance models were examined, including dominant, recessive, additive, and co-dominant models. The dominant model compared carriers of at least one minor allele with homozygous major allele carriers, whereas the recessive model evaluated homozygous minor allele carriers against all other genotypes. The additive model assumed a dose-dependent effect of the minor allele, and the co-dominant model assessed each genotype independently. For SNPs with low genotype counts in specific categories, rare genotypes were combined or excluded in accordance with data protection regulations and to ensure analytical stability.

This study was designed as a hypothesis-driven candidate gene association analysis focusing on CTNNB1. Given the limited number of SNPs examined and the strong biological rationale, formal multiple-testing correction was not applied, and the findings should be interpreted as exploratory.

For transparency, generative artificial intelligence tools were used solely to assist with language refinement and manuscript editing, including the reorganization and clarification of scientific text. These tools did not contribute to study design, data collection, data analysis, data interpretation, or the generation of scientific conclusions. All scientific content and interpretations were determined and verified by the authors.

## 3. Results

A total of 609 patients diagnosed with osteonecrosis of the femoral head (ONFH) and 2436 control subjects were included in the analysis. The ONFH cases included in this analysis primarily represent non-traumatic ONFH, as defined by clinical diagnosis and exclusion of acute traumatic events. The ONFH group comprised 332 males (54.5%) and 277 females (45.5%), while the control group included 1330 males (54.6%) and 1106 females (45.4%). There were no significant differences in sex distribution between the two groups (*p* > 0.999). With respect to age, 112 patients (18.4%) in the ONFH group were aged 40 years or younger, and 497 patients (81.6%) were older than 40 years. The corresponding figures in the control group were 448 individuals (18.4%) aged 40 years or younger and 1988 individuals (81.6%) older than 40 years. No significant difference in age distribution was observed between cases and controls (*p* > 0.999). The mean age was identical in both groups (55.7 ± 15.6 years; *p* = 0.988) (Table 1).

Hardy–Weinberg equilibrium (HWE) was assessed in the control group (Table 2). Fourteen SNPs located within or near the CTNNB1 gene were successfully genotyped. All SNPs showed no substantial deviation from HWE, except rs3774371, which demonstrated a borderline deviation (HWE *p* = 0.029) (Table 2). Among the analyzed variants, two SNPs—rs3774370 and rs11564478—demonstrated statistically significant differences in allele frequency distribution between the ONFH and control groups. The minor allele frequency (MAF) of rs3774370 was 0.0099 in the ONFH group and 0.0189 in the control group (*p* = 0.030), while the MAF of rs11564478 was 0.0099 in cases and 0.0183 in controls (*p* = 0.040). Both SNPs exhibited odds ratios below 1.0, indicating a lower frequency of the minor alleles among ONFH patients. The remaining twelve SNPs did not show significant differences in allele frequencies between cases and controls (Table 2).

The associations between CTNNB1 polymorphisms and ONFH susceptibility were further evaluated under multiple genetic inheritance models using logistic regression analysis (Table 3). For rs3774370, an association with reduced ONFH risk was observed under the dominant model (GG + AG vs. AA), with an adjusted odds ratio of 0.53 (95% confidence interval [CI]: 0.29–0.96, *p* = 0.037). Similarly, rs11564478 showed a protective association under the dominant model (TT + GT vs. GG), yielding an adjusted odds ratio of 0.50 (95% CI: 0.27–0.93, *p* = 0.029). No statistically significant associations were detected for the remaining CTNNB1 SNPs under dominant, recessive, additive, or co-dominant genetic models. For SNPs with low genotype counts in specific categories, rare genotypes were combined or excluded from analysis in accordance with data protection regulations to ensure analytical stability. Accordingly, these variants were not interpreted as independent genetic signals in the present study.

## 4. Discussion

In the present study, we investigated the association between CTNNB1 gene polymorphisms and susceptibility to non-traumatic ONFH using a hospital-based case–control design. Among the fourteen analyzed SNPs, two variants—rs3774370 and rs11564478—demonstrated statistically significant differences in allele frequency between ONFH patients and control subjects. The minor alleles of both SNPs were less frequent in the ONFH group and were associated with reduced disease risk, with odds ratios below 1.0. These protective associations remained significant under dominant genetic models after adjustment, suggesting that CTNNB1 polymorphisms may modulate individual susceptibility to non-traumatic ONFH.

Genetic susceptibility has long been recognized as an important contributor to non-traumatic ONFH, particularly in individuals exposed to well-established environmental risk factors such as corticosteroids and alcohol [10]. Previous studies have identified associations between ONFH and polymorphisms in genes involved in lipid metabolism, vascular regulation, and inflammatory pathways, including RAB40C [46], NOS3 [47], PFKP and GPC6 [46], as well as TNFRSF11A, IL-6, and TNF [49]. However, these findings collectively underscore the complex and polygenic nature of ONFH, as no single genetic variant has demonstrated sufficient predictive capacity or clear translational applicability. Our findings extend this body of evidence by identifying CTNNB1, a key regulator of Wnt/β-catenin signaling, as a novel genetic contributor to ONFH susceptibility.

Polymorphisms in CTNNB1 have been implicated in a wide range of human diseases, particularly malignancies, including prostate, gastric, breast, ovarian, colorectal, and hepatocellular cancers [17]. These associations are thought to arise from altered β-catenin stability, nuclear translocation, or transcriptional activity, leading to dysregulation of downstream target genes. Importantly, synonymous and non-coding variants may influence RNA stability, translational efficiency, and protein folding, thereby exerting functional effects despite not altering amino acid sequences [66]. Such mechanisms provide biological plausibility for the observed associations between CTNNB1 polymorphisms and disease susceptibility.

Beyond its genetic associations, CTNNB1 plays a central role in biological processes directly relevant to non-traumatic ONFH pathogenesis. The Wnt/β-catenin signaling pathway is critical for maintaining the balance between osteogenesis and adipogenesis in mesenchymal stem cells, as well as for regulating angiogenesis within bone tissue [6,60,61,62]. Dysregulation of this pathway has been shown to impair osteogenic differentiation while promoting adipogenesis, a pathological hallmark frequently observed in necrotic femoral heads [61,62]. In addition, β-catenin signaling modulates vascular endothelial function and the expression of pro-angiogenic factors, such as vascular endothelial growth factor, thereby influencing bone perfusion and repair [60]. Experimental studies have demonstrated that suppression of β-catenin activity—such as that induced by ethanol exposure—can exacerbate ONFH progression, whereas pharmacologic activation of the Wnt/β-catenin pathway may mitigate disease development [62,67,68]. These mechanistic insights support the biological relevance of CTNNB1 polymorphisms in non-traumatic ONFH.

In the current era of genome-wide association studies (GWAS), large-scale, hypothesis-free approaches have greatly expanded the identification of genetic loci associated with complex diseases [51]. Nevertheless, GWAS findings often explain only a limited proportion of heritability and frequently identify loci with unclear functional relevance [52]. In contrast, candidate gene studies guided by established disease pathogenesis remain highly valuable [58,59], particularly for conditions such as non-traumatic ONFH, in which the underlying biological mechanisms—vascular compromise, impaired osteogenesis, and aberrant mesenchymal stem cell differentiation—are well characterized. By focusing on CTNNB1, a gene with strong biological plausibility within these pathways, our study complements GWAS approaches and provides mechanistic context that may facilitate interpretation and future translational research.

It should be noted that the two variants showing significant associations, rs3774370 and rs11564478, are low-frequency SNPs located in close physical proximity within the CTNNB1 locus. Therefore, the possibility that these associations reflect linkage disequilibrium rather than independent genetic effects cannot be excluded. Formal linkage disequilibrium metrics (e.g., r^2^ or D’) and conditional or haplotype analyses were not performed in the present study because the very low minor allele frequencies resulted in limited genotype counts, rendering such analyses underpowered and potentially unstable. Future studies with larger sample sizes or independent cohorts will be required to clarify whether these variants represent independent signals or reflect a shared underlying genetic effect.

Several limitations of this study should be acknowledged. First, due to the nature of the available clinical data, we were unable to further stratify non-traumatic ONFH cases according to specific etiologies, such as corticosteroid-associated or alcohol-induced disease. Although both factors represent major and overlapping contributors to non-traumatic ONFH, the lack of etiologic stratification precluded evaluation of exposure-specific gene–environment interactions in the present analysis. This limitation should be considered when interpreting the observed associations. Second, as a case–control study, causal relationships cannot be definitively established, and functional validation experiments will be necessary to elucidate the molecular consequences of the identified SNPs. Third, because multiple SNPs and genetic models were evaluated, the observed associations should be interpreted cautiously and considered exploratory until validated in independent cohorts. In addition, rare variants, structural variants, and regulatory elements within CTNNB1 were not assessed in the present study. Fourth, principal component analysis was not performed to adjust for population stratification. However, the study population was derived from a single medical center and consisted of an ethnically homogeneous Taiwanese population, which reduces—but does not eliminate—the likelihood of major population stratification. Consequently, the findings may not be directly generalizable to populations of different ancestral backgrounds. Despite these limitations, this study provides novel evidence that CTNNB1 polymorphisms are associated with susceptibility to non-traumatic ONFH. By integrating genetic association analysis with established pathogenic mechanisms, our findings contribute to a more refined understanding of ONFH genetics and underscore the continued relevance of pathogenesis-driven genetic studies in the GWAS era.

## 5. Conclusions

In conclusion, this study identified an association between CTNNB1 polymorphisms and susceptibility to non-traumatic osteonecrosis of the femoral head in a Taiwanese population. Two low-frequency variants within the CTNNB1 locus, rs3774370 and rs11564478, were associated with a reduced risk of ONFH, highlighting the potential involvement of the Wnt/β-catenin signaling pathway in disease susceptibility. Given the exploratory nature of this candidate gene analysis, the low minor allele frequencies of the identified variants, and the absence of functional validation, these findings should be interpreted with caution. Nevertheless, by focusing on a biologically relevant pathway, this study underscores the continued value of pathogenesis-driven genetic association studies in the genome-wide association study era. Further investigations in independent cohorts and functional studies will be required to clarify the underlying mechanisms and to determine the broader relevance of CTNNB1 variants in non-traumatic ONFH.

## Figures and Tables

**Table 1:** Baseline demographic characteristics of patients with osteonecrosis of the femoral head (ONFH) and control subjects.

	ONFH Group	Control Group	*p*-Value
Numbers	609	2436	
Gender			>0.999
Male	332 (54.5%)	1330 (54.6%)	
Female	277 (45.5%)	1106 (45.4%)	
Age			>0.999
≤40	112 (18.4%)	448 (18.4%)	
>40	497 (81.6%)	1988 (81.6%)	
Mean (SD)	55.7 (15.6)	55.7 (15.6)	0.988

ONFH: osteonecrosis of the femoral head; SD: standard deviation.

**Table 2:** Genotyping of Catenin Beta-1 (CTNNB1) polymorphisms in patients with osteonecrosis of the femoral head (ONFH) and control subjects.

			MAF				
SNPs	Chr:Position	Allele	ONFH	Control	HWE *p*-Value	OR (95% CI)	*p*-Value
rs117216198	3:41196461	G/A	0.0345	0.0249	0.755	1.40 (0.98, 2.00)	0.062
rs117090459	3:41207590	G/A	0.0272	0.0197	0.153	1.39 (0.93, 2.07)	0.106
rs4016435	3:41208638	T/G	0.0474	0.0496	0.565	0.95 (0.71, 1.28)	0.757
rs2140090	3:41213155	T/G	0.2545	0.2478	0.281	1.04 (0.90, 1.20)	0.630
rs34565533	3:41218911	A/G	0.2545	0.2473	0.481	1.04 (0.90, 1.20)	0.604
rs1798802	3:41220488	A/G	0.3013	0.2967	0.206	1.02 (0.89, 1.17)	0.751
rs3774370 *	**3:41231618**	**G/A**	**0.0099**	**0.0189**	**0.152**	**0.52 (0.28, 0.95)**	**0.030**
rs2276826	3:41234314	G/A	0.0403	0.0349	0.060	1.16 (0.84, 1.60)	0.368
rs3774371	3:41234675	T/A	0.2451	0.2325	0.029	1.07 (0.93, 1.24)	0.357
rs11564465	3:41235549	T/C	0.2479	0.2317	0.133	1.09 (0.94, 1.27)	0.234
rs11564475	3:41238542	G/A	0.1431	0.1414	0.445	1.01 (0.85, 1.21)	0.877
rs11564478 *	**3:41238901**	**T/G**	**0.0099**	**0.0183**	**0.353**	**0.53 (0.29, 0.98)**	**0.040**
rs9850653	3:41251245	T/C	0.2253	0.2177	0.104	1.04 (0.90, 1.22)	0.568
rs1608726	3:41257152	A/G	0.4557	0.4356	0.679	1.08 (0.96, 1.23)	0.207

SNPs: single-nucleotide polymorphisms; Chr: chromosome; MAF: minor allele frequency; ONFH: osteonecrosis of the femoral head; HWE: Hardy–Weinberg equilibrium; OR: odds ratio; CI: confidence interval. * Bold values indicate SNPs showing statistically significant associations with ONFH (*p*-Value < 0.05).

**Table 3:** Association between Catenin Beta-1 (CTNNB1) polymorphisms and the risk of osteonecrosis of the femoral head (ONFH) under different genetic models.

					Crude Analysis		Adjusted Analysis	
SNPs	Model	Genotype	Case	Control	OR (95% CI)	*p*-Value	OR (95% CI)	*p*-Value
rs117216198	Dominant *	AA	568	2314	1.00 (ref.)		1.00 (ref.)	
		GG + AG *	40	118	1.38 (0.95, 2.00)	0.087	1.38 (0.95, 2.00)	0.088
rs117090459	Dominant *	AA	574	2340	1.00 (ref.)		1.00 (ref.)	
		GG + AG *	33	94	1.43 (0.95, 2.15)	0.084	1.46 (0.97, 2.19)	0.071
rs4016435	Dominant *	GG	546	2167	1.00 (ref.)		1.00 (ref.)	
		TT + GT *	55	233	0.94 (0.69, 1.28)	0.679	0.95 (0.69, 1.29)	0.722
rs2140090	Co-dominant	GG	336	1384	1.00 (ref.)		1.00 (ref.)	
		TT	236	892	0.97 (0.67, 1.42)	0.877	0.97 (0.67, 1.42)	0.886
		GT	37	157	1.09 (0.90, 1.31)	0.366	1.09 (0.91, 1.32)	0.354
	Dominant	GG	336	1384	1.00 (ref.)		1.00 (ref.)	
		TT + GT	273	1049	1.07 (0.90, 1.28)	0.446	1.07 (0.90, 1.29)	0.431
	Recessive	TT	236	892	1.00 (ref.)		1.00 (ref.)	
		GT + GG	373	1541	0.94 (0.65, 1.36)	0.733	0.94 (0.65, 1.36)	0.738
	Additive	-	-	-	1.04 (0.90, 1.20)	0.632	1.04 (0.90, 1.20)	0.616
rs34565533	Co-dominant	GG	336	1386	1.00 (ref.)		1.00 (ref.)	
		AA	236	892	0.98 (0.67, 1.43)	0.910	0.98 (0.67, 1.43)	0.918
		GA	37	156	1.09 (0.91, 1.32)	0.358	1.09 (0.91, 1.32)	0.346
	Dominant	GG	336	1386	1.00 (ref.)		1.00 (ref.)	
		AA + GA	273	1048	1.07 (0.90, 1.28)	0.430	1.08 (0.90, 1.29)	0.416
	Recessive	AA	236	892	1.00 (ref.)		1.00 (ref.)	
		GA + GG	373	1542	0.94 (0.65, 1.37)	0.763	0.95 (0.65, 1.37)	0.767
	Additive	-	-	-	1.04 (0.90, 1.20)	0.605	1.04 (0.90, 1.20)	0.590
rs1798802	Co-dominant	GG	298	1207	1.00 (ref.)		1.00 (ref.)	
		AA	255	1007	1.04 (0.76, 1.43)	0.808	1.05 (0.76, 1.45)	0.771
		GA	56	218	1.03 (0.85, 1.24)	0.791	1.03 (0.85, 1.24)	0.769
	Dominant	GG	298	1207	1.00 (ref.)		1.00 (ref.)	
		AA + GA	311	1225	1.03 (0.86, 1.23)	0.758	1.03 (0.86, 1.23)	0.728
	Recessive	AA	255	1007	1.00 (ref.)		1.00 (ref.)	
		GA + GG	354	1425	1.03 (0.76, 1.40)	0.858	1.04 (0.76, 1.41)	0.826
	Additive	-	-	-	1.02 (0.89, 1.17)	0.752	1.03 (0.89, 1.18)	0.715
rs3774370	Dominant *	AA	596	2342	1.00 (ref.)		1.00 (ref.)	
		GG + AG *	12	91	**0.52 (0.28, 0.95)**	**0.032**	**0.53 (0.29, 0.96)**	**0.037**
rs2276826	Dominant *	AA	559	2267	1.00 (ref.)		1.00 (ref.)	
		GG + AG *	49	167	1.19 (0.85, 1.66)	0.304	1.19 (0.85, 1.66)	0.315
rs3774371	Co-dominant	AA	346	1433	1.00 (ref.)		1.00 (ref.)	
		TT	223	861	1.14 (0.78, 1.68)	0.492	1.14 (0.78, 1.68)	0.490
		AT	37	134	1.07 (0.89, 1.29)	0.465	1.07 (0.89, 1.30)	0.459
	Dominant	AA	346	1433	1.00 (ref.)		1.00 (ref.)	
		TT + AT	260	995	1.08 (0.90, 1.30)	0.390	1.08 (0.90, 1.30)	0.385
	Recessive	TT	223	861	1.00 (ref.)		1.00 (ref.)	
		AT + AA	383	1567	1.11 (0.76, 1.62)	0.576	1.11 (0.76, 1.62)	0.575
	Additive	-	-	-	1.07 (0.93, 1.24)	0.358	1.07 (0.93, 1.24)	0.354
rs11564465	Co-dominant	CC	343	1432	1.00 (ref.)		1.00 (ref.)	
		TT	224	853	1.18 (0.81, 1.73)	0.383	1.19 (0.81, 1.73)	0.381
		CT	38	134	1.10 (0.91, 1.32)	0.339	1.10 (0.91, 1.33)	0.334
	Dominant	CC	343	1432	1.00 (ref.)		1.00 (ref.)	
		TT + CT	262	987	1.11 (0.93, 1.33)	0.263	1.11 (0.93, 1.33)	0.260
	Recessive	TT	224	853	1.00 (ref.)		1.00 (ref.)	
		CT + CC	381	1566	1.14 (0.79, 1.66)	0.482	1.14 (0.79, 1.66)	0.481
	Additive	-	-	-	1.09 (0.94, 1.26)	0.236	1.09 (0.94, 1.27)	0.233
rs11564475	Co-dominant	AA	441	1802	1.00 (ref.)		1.00 (ref.)	
		GG	160	569	0.48 (0.22, 1.07)	0.073	0.49 (0.22, 1.08)	0.078
		AG	7	59	1.15 (0.94, 1.41)	0.182	1.14 (0.93, 1.40)	0.214
	Dominant	AA	441	1802	1.00 (ref.)		1.00 (ref.)	
		GG + AG	167	628	1.09 (0.89, 1.33)	0.415	1.08 (0.88, 1.32)	0.462
	Recessive	GG	160	569	1.00 (ref.)		1.00 (ref.)	
		AG + AA	448	1861	0.47 (0.21, 1.03)	0.059	0.48 (0.22, 1.05)	0.065
	Additive	-	-	-	1.01 (0.85, 1.21)	0.878	1.01 (0.84, 1.21)	0.922
rs11564478	Dominant *	GG	596	2343	1.00 (ref.)		1.00 (ref.)	
		TT + GT *	12	89	**0.53 (0.29, 0.97)**	**0.041**	**0.50 (0.27, 0.93)**	**0.029**
rs9850653	Co-dominant	CC	370	1493	1.00 (ref.)		1.00 (ref.)	
		TT	202	822	1.22 (0.83, 1.80)	0.316	1.23 (0.83, 1.81)	0.309
		CT	36	119	0.99 (0.82, 1.20)	0.931	0.99 (0.82, 1.20)	0.931
	Dominant	CC	370	1493	1.00 (ref.)		1.00 (ref.)	
		TT + CT	238	941	1.02 (0.85, 1.22)	0.827	1.02 (0.85, 1.23)	0.824
	Recessive	TT	202	822	1.00 (ref.)		1.00 (ref.)	
		CT + CC	406	1612	1.22 (0.83, 1.80)	0.301	1.23 (0.84, 1.81)	0.294
	Additive	-	-	-	1.04 (0.90, 1.21)	0.571	1.05 (0.90, 1.21)	0.565
rs1608726	Co-dominant	GG	175	791	1.00 (ref.)		1.00 (ref.)	
		AA	313	1161	1.14 (0.88, 1.48)	0.306	1.15 (0.89, 1.49)	0.286
		GA	121	478	1.22 (0.99, 1.50)	0.060	1.21 (0.98, 1.49)	0.073
	Dominant	GG	175	791	1.00 (ref.)		1.00 (ref.)	
		AA + GA	434	1639	1.20 (0.99, 1.45)	0.070	1.19 (0.98, 1.45)	0.079
	Recessive	AA	313	1161	1.00 (ref.)		1.00 (ref.)	
		GA + GG	296	1269	1.01 (0.81, 1.27)	0.913	1.02 (0.82, 1.28)	0.834
	Additive	-	-	-	1.08 (0.96, 1.23)	0.211	1.09 (0.96, 1.23)	0.202

ONFH: osteonecrosis of the femoral head; SNPs: single-nucleotide polymorphisms; OR: odds ratio; CI: confidence interval; NA, not applicable. * When the number of individuals in certain genotype categories was less than 3 (e.g., GG or AG in rs117216198), those categories were either merged with other genotypes or excluded from analysis in accordance with Taiwan government regulations to protect participant privacy. For SNPs marked with an asterisk (*), only the dominant genetic model is presented, where rare genotypes were combined (e.g., GG + AG vs. AA), and other models were not shown due to limited sample size and unstable estimates. Bold values indicate SNPs showing statistically significant associations with ONFH (*p*-Value < 0.05).

## Data Availability

The data presented in this study are available on reasonable request from the corresponding author because the dataset contains sensitive human genetic and clinical information derived from hospital-based records. In accordance with institutional regulations, ethical approval requirements, and data protection policies, the raw data cannot be made publicly available.

## Abbreviations

The following abbreviations are used in this manuscript:

ONFH	Osteonecrosis of the femoral head
SNP	Single-nucleotide polymorphism
GWAS	Genome-wide association study
CTNNB1	Catenin beta-1
CMUH	China Medical University Hospital
ICD-9	International Classification of Diseases, Ninth Revision
ICD-10	International Classification of Diseases, Tenth Revision
TWBv2	Taiwan Biobank version 2
MAF	Minor allele frequency
OR	Odds ratio
CI	Confidence interval
HWE	Hardy–Weinberg equilibrium
MSC	Mesenchymal stem cell
