# Correction: Lai et al. Association Between Catenin Beta-1 (CTNNB1) Gene Polymorphisms and Non-Traumatic Osteonecrosis of the Femoral Head (ONFH). *Curr. Issues Mol. Biol.* 2026, *48*, 164

**DOI:** 10.3390/cimb48050504

**Published:** 2026-05-14

**Authors:** I-Chang Lai, De-Yi Liu, Shih-Chan Hsu, Shu-Jui Kuo

**Affiliations:** 1 Department of Education, China Medical University Hospital, Taichung 404327, Taiwan; lai.i.chang.58@gmail.com (I.-C.L.); deyil845@gmail.com (D.-Y.L.); 2 School of Medicine, China Medical University, Taichung 404328, Taiwan; jamieee0424@gmail.com; 3 Department of Orthopedic Surgery, China Medical University Hospital, Taichung 404327, Taiwan

## Additional Affiliations

In the published article [1], there was an error in the author affiliations. In the original publication, the affiliations of Shih-Chan Hsu and the corresponding author were inaccurately represented due to an oversight during the submission and proofreading process. This correction ensures accurate representation of Shih-Chan Hsu’s institutional affiliation, as well as the corresponding author’s academic and clinical roles. The authors state that the scientific conclusions are unaffected. This correction was approved by the Academic Editor. The original publication has also been updated.

## References

[1] Lai I.-C., Liu D.-Y., Hsu S.-C., Kuo S.-J. (2026). Association Between Catenin Beta-1 (CTNNB1) Gene Polymorphisms and Non-Traumatic Osteonecrosis of the Femoral Head (ONFH). Curr. Issues Mol. Biol..

